# Comparative Immunogenicity of HIV-1 Clade C Envelope Proteins for Prime/Boost Studies

**DOI:** 10.1371/journal.pone.0012076

**Published:** 2010-08-11

**Authors:** Douglas H. Smith, Peggy Winters-Digiacinto, Misrach Mitiku, Sara O'Rourke, Faruk Sinangil, Terri Wrin, David C. Montefiori, Phillip W. Berman

**Affiliations:** 1 VaxGen, Inc., Brisbane, California, United States of America; 2 Baskin School of Engineering, University of California Santa Cruz, Santa Cruz, California, United States of America; 3 Global Solutions For Infectious Diseases, South San Francisco, California, United States of America; 4 Monogram Biosciences, South San Francisco, California, United States of America; 5 Duke University Medical School, Durham, North Carolina, United States of America; University of California San Francisco, United States of America

## Abstract

**Background:**

Previous clinical efficacy trials failed to support the continued development of recombinant gp120 (rgp120) as a candidate HIV vaccine. However, the recent RV144 HIV vaccine trial in Thailand showed that a prime/boost immunization strategy involving priming with canarypox vCP1521 followed by boosting with rgp120 could provide significant, although modest, protection from HIV infection. Based on these results, there is renewed interest in the development of rgp120 based antigens for follow up vaccine trials, where this immunization approach can be applied to other cohorts at high risk for HIV infection. Of particular interest are cohorts in Africa, India, and China that are infected with clade C viruses.

**Methodology/Principal Findings:**

A panel of 10 clade C rgp120 envelope proteins was expressed in 293 cells, purified by immunoaffinity chromatography, and used to immunize guinea pigs. The resulting sera were collected and analyzed in checkerboard experiments for rgp120 binding, V3 peptide binding, and CD4 blocking activity. Virus neutralization studies were carried out with two different assays and two different panels of clade C viruses. A high degree of cross reactivity against clade C and clade B viruses and viral proteins was observed. Most, but not all of the immunogens tested elicited antibodies that neutralized tier 1 clade B viruses, and some sera neutralized multiple clade C viruses. Immunization with rgp120 from the CN97001 strain of HIV appeared to elicit higher cross neutralizing antibody titers than the other antigens tested.

**Conclusions/Significance:**

While all of the clade C antigens tested were immunogenic, some were more effective than others in eliciting virus neutralizing antibodies. Neutralization titers did not correlate with rgp120 binding, V3 peptide binding, or CD4 blocking activity. CN97001 rgp120 elicited the highest level of neutralizing antibodies, and should be considered for further HIV vaccine development studies.

## Introduction

The development of a vaccine to prevent HIV infection is a global health priority. After more than 25 years of research, a modest level of protection was recently achieved in humans in a Phase 3 HIV-1 vaccine trial (RV144) involving more than 16,000 subjects in Thailand [1]. The RV144 study entailed priming immunizations with a recombinant canarypox vector (vCP1521) containing HIV *env* and *gag* genes to elicit cellular immune responses [2]. This was followed by booster immunizations with a bivalent rgp120 subunit vaccine, AIDSVAX B/E [3]–[5], to elicit antibody responses. The results of this trial were surprising because previous studies showed that these vaccines, given alone, were unable to elicit consistent T cell responses [6] or protective antibody responses [7], [8]. Indeed, as a result of these studies, product development efforts on both rgp120-based vaccines and canarypox virus-based vaccines were largely discontinued, and efforts were refocused on adenovirus-based vaccines [9] and trimeric envelope glycoprotein vaccines [10], [11]. However, the results from the RV144 trial have rekindled interest in both rgp120 subunit vaccines and canarypox virus vectors. There is now strong interest in confirmatory clinical studies with similar rgp120 vaccines and recombinant pox virus vectors designed for regions of the world where different clades (subtypes) of HIV are in circulation. Because it is desirable to match the genetic clade of vaccine immunogens to the viruses circulating in clinical cohorts, the AIDSVAX B/E rgp120 vaccine used in Thailand in the RV144 trial is considered inappropriate for clinical trials in Africa, India, or China, where clade C viruses account for the majority of new infections (UNAIDS, http://www.unaids.org). As a consequence, there is new interest in clade C rgp120 vaccines. These studies represent the only comparative immunogenicity of clade C rgp120s with diverse amino acid sequences, prepared in a manner identical to the envelope immunogens used in the AIDSVAX B/E vaccine. Here we compare the magnitude and specificity of functionally significant antibody responses to clade C rgp120 antigens. These results may be useful in the selection of clade C vaccine antigens that could potentially be used in clinical development studies designed to repeat the RV144 prime/boost immunization regimen.

## Results

In considering the development of clade C vaccine immunogens, we wanted to encompass the range of sequence variation found in clade C envelope proteins from different regions of the world. For this purpose, we assembled a collection of ten clade C envelope genes with diverse sequences. The final collection included one isolate from Zambia, two each from China, India, and Tanzania, and three from South Africa (Table 1). An alignment of the amino acid sequences of these strains is provided in supplemental Figure S1. A pairwise comparison of protein sequence homology is provided in Table 2. A phylogenetic analysis of the HIV envelope protein rgp120 used in this study along with a diverse collection of clade C virus reference sequences from the panel of Li et al. [12] and the Los Alamos HIV sequence database (www.hiv.lanl.gov/.webloc) are provided in Figure S2. It can be seen that the viruses selected for protein expression studies are well distributed throughout the phylogeny and do not form unrepresentative clusters. However, the relevance of phylogenetic trees with respect to protective immune responses is uncertain. For this reason we were also interested in carrying out phylogenetic analysis of domains containing important neutralizing epitopes such as the V3 domain [13]. The results provided in Figure S3 and Table S1 show a high level of V3 sequence diversity within the panel of viruses studied.

**Table 1:** Listing of clade C viruses.

Strain	Year collected	Origin
CN97001	1997	China
CN98005	1998	China
IN98025	1998	India
IN98026	1998	India
TZ97005	1997	Tanzania
TZ97008	1997	Tanzania
ZA97002	1997	South Africa
ZA97010	1997	South Africa
ZA97012	1997	South Africa
ZM651	1996	Zambia

Clade C envelope genes were cloned from HIV infected cell lysates provided by the joint United Nations program on HIV/AIDS and the National Institutes of Allergy and Infectious Diseases, NIH. Viruses were selected on the basis of geographical diversity as well as variation in V3 domain sequences.

**Table 2:** Pairwise comparison of clade C rgp120 sequences.

	ZM651	IN98025	IN98026	TZ97008	ZA97002	TZ97005	CN98005	ZA97010	CN97001	ZA97012
ZM651	*	78	77	77	78	79	77	77	77	75
IN98025		*	84	77	78	79	84	78	83	77
IN98026			*	79	78	80	85	79	85	78
TZ97008				*	79	79	78	79	78	78
ZA97002					*	78	78	78	77	78
TZ97005						*	79	77	80	77
CN98005							*	77	89	77
ZA97010								*	78	81
CN97001									*	77
ZA97012										*

Amino acid sequences were deduced from the DNA sequences of the 10 clade C rgp120 genes. Pairs of sequences were aligned, and the percent identity between each pair determined using the MacVector Sequence Analysis package (Accelrys Inc., San Diego, CA).

**Figure 1:**
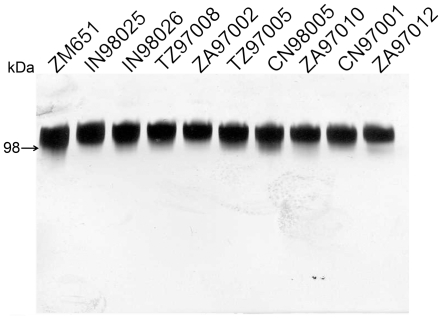
SDS-PAGE showing purified subtype C rgp120 antigens. 10 different subtype C rgp120 isolate proteins were expressed in 293 T cells and purified. 150ng of purified protein was run on SDS PAGE gel and visualized by silver staining. Arrow indicates mobility of molecular weight standard.

All 10 envelope genes were then expressed in mammalian cells and purified for immunization studies. The mobility and purity of the purified envelope proteins by SDS-polyacrylamide gel chromatography is shown in Figure 1. The envelope proteins were found to be free of proteolysis products and greater than 98% pure as indicated by silver staining. Groups of 7 guinea pigs were immunized with each of the 10 clade antigens as described in the Materials and Methods section. In addition, two groups of animals were immunized with MN-rgp120 and A244-rgp120 that were included in the AIDSVAX B/E vaccine [3], [4]. This vaccine was used in the Phase I/II and Phase III clinical trials alone [5], [7] and in connection with vCP1521 in the RV144 clinical trial [1].

Initially, we measured the binding of the resulting antibodies to homologous and heterologous rgp120 by the same ELISA assay (supplemental Figure S4) described previously [14]. We found that all of the antigens were immunogenic and elicited high levels of antibodies similar to those described previously [15]. We then examined the cross reactivity of each serum pool with rgp120s from clade C (ZM651), clade B (MN), clade E (CM244), and clade D (Z6) strains of HIV (Figure S4). Each of the clade C serum pools exhibited a high level of cross reactivity with envelope proteins from different clades; however, the magnitude of the immune response to the ZM651 protein was somewhat greater than that of the other immunogens. This level of cross reactivity was not unexpected, since previous studies also showed a high degree of cross reactivity of antisera to rgp120 with envelope proteins from CCR5 and CXCR4 viruses of different clades [3], [16].

**Figure 2:**
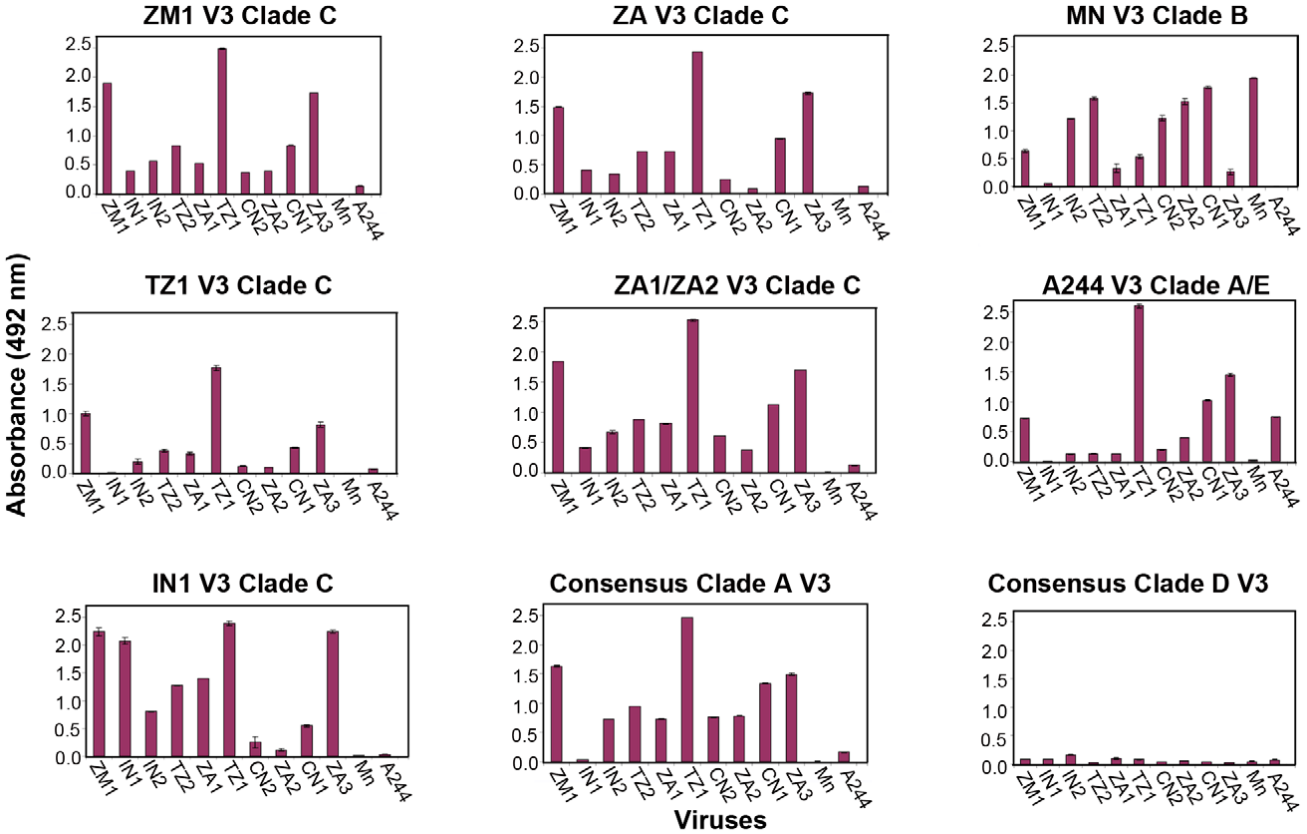
Binding of antisera against V3 peptides from subtypes C, A, B, E and D envelope proteins. ELISA plates were coated with 23-mer V3 peptides corresponding to the subtype C isolates, as well as the sequence for V3 domain peptides from subtype A, B, E (crf A/E), and D envelope glycoproteins. Antibody binding was measured at a 1∶500 serum dilution as described previously [14]. The data bars correspond to the following strains: ZM1, ZM651; IN1, IN98025; IN2, IN98026; TZ2, TZ97008; ZA1, ZA97002; TZ1, TZ97005; CN2, CN98005; ZA2, ZA97010; CN1, CN97001; ZA3, ZA97012.

We next examined the formation of antibodies to the V3 domain, which is known to contain important neutralizing epitopes [17]. The sequence of the synthetic peptides used in these assays is provided in supplemental Table S1. Large differences were observed in the magnitude and specificity of the V3 antibody response elicited by different clade C immunogens (Figure 2). For example, antibodies to both the ZM651 and TZ97002 envelope proteins elicited a high level of cross reactivity with the V3 peptides from clade C and clade A peptides, but poor reactivity with the clade D V3 peptide. Many of the clade C pools reacted with the clade B V3 peptide and a few reacted with the clade E V3 peptide. Significantly, antisera to MN-rgp120 and A244-rgp120 exhibited poor cross reactivity with clade C V3 peptides, demonstrating that while antibodies to clade C immunogens are able to bind to clade B and E V3 peptides, the reverse is not true. These studies suggest that immunization with vaccines containing MN- and A244-rgp120s (e.g. AIDSVAX B/E) would not be effective in eliciting antibodies to the V3 domain of clade C viruses.

We next examined the ability of the clade C sera to inhibit the binding of rgp120 to soluble CD4 (Table 3). All of the envelopes tested elicited antibodies able to block the binding of rgp120 to CD4. As expected, the highest level of inhibition was usually, but not always, observed with homologous protein. However, there appeared to be significant differences in the ability of different immunogens to elicit CD4 blocking antibodies. For example, the ZM651, IN98025, and IN98026 sera were less effective in inhibiting the binding of heterologous envelopes to CD4 than sera to most of the other antigens tested.

**Table 3:** Blocking of CD4 binding to rgp120 by antisera against subtype C rgp120.

	Antiserum									
Coat	ZM651	IN98025	IN98026	TZ97008	ZA97002	TZ97005	CN98005	ZA97010	CN97001	ZA97012
ZM651	***67***	39	55	71	76	83	67	77	77	54
IN98026	46	50	***80***	69	70	77	69	73	74	61
TZ97008	26	14	35	***69***	60	61	53	66	62	52
ZA97002	64	63	83	83	***84***	77	66	79	75	70
TZ97005	47	27	46	56	62	***77***	50	56	57	51
CN98005	47	39	55	59	64	68	***76***	68	70	57
ZA97010	32	30	48	72	72	64	58	***91***	70	62
CN97001	41	30	45	64	71	74	64	80	***96***	62
ZA97012	47	37	53	75	73	69	64	75	71	***76***

The ability of antibodies to inhibit the binding of CD4-IgG to rgp120 was measured using an assay similar to that described previously [14]. Briefly, rgp120 was captured onto microtiter dishes. Sera were added at a 1∶200 dilution in duplicate and incubated 2 hr, and soluble CD4 was then added without washing. The amount of bound CD4 with and without added anti-rgp120 sera was monitored by the binding of HRP-labeled mouse monoclonal antibody to CD4. Results using rgp120 from the IN98025 isolate were not available due to the inability of this envelope protein to bind CD4-IgG.

The ability of antisera from all 10 clade C antigens to neutralize clade B and C viruses was measured in two different laboratories using two different panels of viruses (Tables 4 and 5). In the first assay (Table 4), pseudotype viruses were prepared from a panel of 10 heterologous clade C viruses and 4 clade B viruses, and used to infect U87 cells transfected with CD4 and chemokine receptors (U87 pseudotype assay)[18], [19]. Interestingly, 8 of the 10 clade C sera pools showed significant neutralization against laboratory-adapted clade B viruses (e.g., MN and SF162) ,whereas the prototypic clinical isolate, JRCSF, was resistant to neutralization. This result confirmed the high level of cross reactivity of clade C antisera against clade B viral proteins. When the neutralizing activity against clade C clinical isolates was examined, significant differences were seen among the sera elicited by different antigens. For example, the sera to the ZM651, IN98025, and ZA97012 envelopes showed the weakest neutralization titers and were unable to neutralize any of the clade C viruses in the panel. In contrast, the sera raised against the CN97001 and IN98026 envelope proteins neutralized multiple clade C viruses. While the neutralization titers to these clade C envelopes were low, so were the neutralization titers obtained with the positive control serum, N16, from an HIV infected individual. This serum is known to possess high levels of broadly neutralizing antibodies [20]. Thus the viruses in this panel for the most part represented difficult to neutralize viruses. The inclusion of the aMLV virus in the U87 pseudotype assay provided a reliable control, demonstrating that the neutralization activity observed was not an artifact of a non-specific immune response to viral membranes.

**Table 4:** Neutralization of clade C and clade B viruses in the U87 pseudotype neutralization assay by antibodies to rgp120.

		Antisera										
Virus	Clade	ZM651	IN98025	IN98026	TN97008	ZA97002	TZ97005	CN98005	ZA97010	CN97001	ZA97012	N16
21068	C	-	-	27	12	21	13	-	-	50	-	150
92ZW101	C	-	-	24	-	-	12	-	-	66	-	233
93IN101	C	-	-	18	-	12	-	-	-	-	-	37
93IN999	C	-	-	-	-	-	-	-	-	-	-	72
93MW960	C	-	-	33	20	21	-	44	-	79	-	270
97ZA102	C	-	-	-	-	-	-	-	-	-	-	45
98BR004	C	-	-	-	-	-	-	-	-	-	-	100
98CN006	C	-	-	18	-	14	14	-	-	42	-	116
98CN009	C	-	-	19	-	-	-	-	-	-	-	60
98IN022	C	-	-	95	55	61	17	261	-	345	38	73
BAL	B	-	-	33	52	13	-	68	-	430	-	683
JRCSF	B	-	-	-	-	-	-	-	-	-	-	211
MN	B	-	-	1882	1669	355	202	2324	796	8863	36	3798
NL43	B	-	-	84	161	282	159	69	66	170	79	1510
SF162	B	183	-	3457	6669	579	1875	4403	3044	13437	197	7358
aMLV	B	<10	11	<10	<10	<10	<10	14	20	<10	<10	<10

Virus neutralization titers were measured in the U87 pseudotype virus neutralization assay [18], [19]. Virus designations include the country of origin as follows: BR, Brazil; CN, China; IN, India; MW, Malawi; SA, South Africa; ZA, South Africa; ZM, Zambia; ZW, Zimbabwe. ND, indicates not done. The neutralizing antibody titer (IC50) is defined as the reciprocal of the plasma dilution that produces a 50% inhibition in target cell infection. A dash indicates no significant neutralization. For this assay, virus neutralization was considered significant if the neutralization titers were at least 3-fold greater than those observed against the control pseudotype virus, aMLV. The positive control was an HIV+ sera (N16).

**Table 5:** Neutralization of clade C and B viruses in the TZM-bl neutralization assay by antibodies to rgp120.

		Antisera											
Virus	Clade	ZM651	N98025	IN98026	TN97008	ZA97002	TZ97005	CN98005	ZA97010	CN97001	ZA97012	NGPS	4E10
SA123.6	C	-	-	-	-	-	62	87	-	103	-	<20	0.2
SA151.2	C	-	-	-	-	-	-	-	-	-	-	<20	0.4
SA156.12	C	-	83	77	-	-	-	-	-	-	-	<20	0.43
SA172.17	C	69	64	146	88	86	117	-	-	160	-	24	0.03
ZM233M.PB6	C	-	-	98	-	-	-	-	-	133	-	22	0.61
ZM197M.PB7	C	-	-	-	-	-	-	-	-	-	-	<20	0.1
MN	B	-	-	2958	2935	626	-	2120	1357	38225	-	<20	0.04
SF162.LS	B	319	-	3536	6714	517	1351	2671	3320	12705	89	<20	0.40

Virus neutralization titers were measured by the TZM-bl neutralization assay [28]. Virus designations include the country of origin as follows: BR, Brazil; CN, China; IN, India; MW, Malawi; SA, South Africa; ZA, South Africa; ZM, Zambia; ZW, Zimbabwe. ND, indicates not done. The neutralizing antibody titer (IC50) is defined as the reciprocal of the plasma dilution that produces a 50% inhibition in target cell infection. A dash indicates no significant neutralization. For this assay, neutralization titers were considered significant if they were 3-fold greater than the titers obtained for each virus pseudotype with normal guinea pig serum (NGPS). The positive control was the 4E10 broadly neutralizing monoclonal antibody.

The same sera were then tested in a second assay (TZM-bl assay) that included 6 clade C viruses and 2 clade B viruses. The results obtained in this TZM-bl assay (Table 5) were qualitatively similar to results obtained in the U87 pseudotype neutralization assay described above. We observed that most of the clade C sera were able to neutralize the clade B, laboratory adapted isolates, MN and SF162. Both assays showed that the CN97001 and IN98026 sera possessed the highest levels of neutralizing activity, whereas the ZM651, IN98025 and ZA97012 sera exhibited the lowest level of neutralizing activity.

## Discussion

These studies demonstrated that envelope proteins from clade C virus were all comparably immunogenic as measured by rgp120 binding; however, some were better than others in eliciting antibodies able to bind to the V3 domain, or block the binding of CD4 to rgp120. Most of the sera to clade C viruses were able to neutralize tier 1 strains of clade B viruses, thus demonstrating some level of cross clade neutralizing activity. This activity may well be attributable to antibodies to the V3 domain of clade C antigens which exhibited significant cross reactivity with clade B V3 domain peptides. It is well known that antibodies to the V3 domain are particularly effective in neutralizing tier 1 viruses. As has been described previously, there was poor neutralization of clinical isolates of HIV-1. This continues to be a problem with all of the candidate HIV vaccines tested to date [21], [22]. Although it has long been speculated that that trimeric forms of HV envelope proteins would be better than monomeric rgp120 with respect to eliciting antibodies able to neutralize clinical isolates, the results to date have been equivocal [23].

Overall the antibody responses strains such as CN97001, and IN98026 were similar to those seen with the envelope proteins contained in the AIDSVAX B/E vaccine used in the RV144 clinical trial. However, other strains (e.g. ZM651, IN98025, ZA97010, ZA97012) were significantly less effective in eliciting neutralizing antibodies. We did not observe any significant correlation between antibodies able to neutralize primary isolates and any of the antibody binding assays. Multiple sera that possessed significant CD4 blocking titers failed to exhibit virus neutralization; demonstrating that CD4 blocking antibodies to rgp120 are not a correlate of virus neutralization. Thus, CD4 blocking antibodies elicited against gp120 immunogens appear to differ from CD4 blocking antibodies present in sera from HIV-infected individuals, where immunoadsorbtion studies have shown that a significant proportion of broadly neutralizing antibodies possess CD4 blocking activity [24], [25]. Whether this difference can be attributed to differences in antibody avidity or antibody specificity remains to be determined. Based on the main criteria used to select the immunogens included in the AIDSVAX B/E vaccine used in the RV144 trial (e.g. immunogenicity, neutralizing activity, and common sequence polymorphisms at neutralizing sites), rgp120 from the CN97001 strain of HIV-1, would be our recommendation for a clade C immunogen to include in a subunit boost in studies designed repeat the RV144 immunization regimen.

However, we don't know if *in vitro* neutralization is a correlate of protective immunity in humans. It is possible that antibodies with activities distinct from those measured in these studies, might be important for protection *in vivo*. Preliminary analysis of the results of the RV144 trial has indicated that protection was achieved in the absence of high titers of neutralizing antibodies (D. Montefiori, oral presentation, HVTN Full Group Meeting, Washington DC, May 4–6, 2010). This result suggests that neutralization titers lower than those that we are able to measure in the TZM-bl assay, or that other types of antibody responses, such as those that mediate antibody dependent cytotoxicity, or reduce virus mobility at mucosal surfaces, might be better correlates of protective immunity. However, until definitive results are available, the correlates of protection remain undefined. The proteins described in this paper provide the only comparative immunogenicity study of clade C rgp120 vaccine antigens constructed in the same manner as the clade B and clade E antigens used for booster immunizations in the RV144 trial. Our results provide a rationale for the selection of immunogens to be used in optimizing prime/boost immunization regimens. Further studies focusing on reactivity with viruses from specific clinical cohorts in which RV144 follow-up trials would be carried out might provide further rationale for the selection of specific immunogens.

## Materials and Methods

### Production of antigens

A collection of ten clade C envelope genes with diverse sequences was assembled for this study. Nine of these were PCR amplified from infected cell lysates provided by Dr. S. Osmanov (WHO-UNAIDS, Geneva, Switzerland) and one (ZM651) was provided by Dr. F. Gao (Duke University, Durham, NC). In order to produce antigens homologous to those used in the RV144 vaccine trial, codon optimized rgp120 sequences were expressed as fusion proteins where the signal sequence and 12 amino acids from the mature N-terminus of rgp120 were deleted, and replaced with the signal sequence and 27 N-terminal amino acids of the mature form of herpes simplex virus type 1 glycoprotein D (gD-1) as described previously [26]. The resulting constructs were cloned into a pCI based expression vector (Promega Corp, Madison, WI) and expressed in Lipofectamine Plus (Invitrogen, Carlsbad, CA) transfected 293 cells. Recombinant envelope proteins were purified from growth conditioned cell culture media and subjected to immunoaffinity chromatography, using a monoclonal antibody specific for the HSV-1 flag epitope.

### Guinea pig immunizations

Immunization studies (approval number 10245) were carried out at Covance Research Products (Denver, PA), an AAALAC accredited organization (OLAW assurance number:A3850-01; USDA research registration number:23-R-007). The study was reviewed and approved by the CRP's Institutional Animal Care and Use Committee (IACUC). Each purified protein was then used to immunize groups of 6 guinea pigs. Each animal received 10µg of rgp120 on Days 0, 14, and 42. All antigens were formulated in QS21 adjuvant that had previously demonstrated a substantial dose-sparing ability as well as eliciting a strong immune response [15]. Sera collected 2 weeks after the final boost from each group were pooled and tested in antibody binding assays.

### Antibody binding assays

Antibody binding to rgp120 from homologous and heterologous rgp120 by ELISA as described previously [5], [7], [14]. Antibody binding assays were carried out with pooled sera from the 7 animals in each group immunized with rgp120 from different strains of HIV. Antibody binding to synthetic peptides from the V3 domain of rgp120 was carried out as described previously [7], [27]. A listing of the V3 peptides used in this study is provided in Table S1. The ability of anti-rgp120 antibodies to block the binding of rgp120 to CD4 from homologous and heterologous strains of virus was measured in an ELISA assay similar to that described previously [27].

### Virus neutralization assays

The ability of antibodies to rgp120 to neutralize various strains of HIV was measured in two different assays that made use of pseudotype viruses. The first assay (U87 pseudotype assay) was performed by Monogram Biosciences (S. San Francisco, CA) and utilized a panel of 10 heterologous clade C viruses and 4 clade B viruses. In this assay, pseudotype viruses prepared in 293 cells were used to infect U87 cells transfected with CD4 and either the CXCR4 or CCR5 chemokine receptors [18], [19]. The second assay (TZM-bl assay) was carried out by Dr. D. Montefiori (Duke Medical School) and utilized the TZM-bl assay format [12], [28]. This assay included 6 clade C viruses and 2 clade B viruses.

### Phylogenetic analysis

Phylogenetic trees were constructed in EMBOSS http://www.ebi.ac.uk .ac.uk using the neighbor joining method [29] by percentage identity, on sequences pre-aligned using the MAFFT algorithm [30].

## Supporting Information

Figure S1 Alignment of subtype C gp120 amino acid sequences. Amino acid sequences were deduced from the DNA sequences of the 10 subtype C gp120 genes. The sequences were aligned using the MacVector Sequence Analysis package (Accelrys Inc. San Diego, CA). The alignment was then edited manually to align significant features, e.g. a pair of conserved N-linked glycosylation sites in the V1 hypervariable region. Bases that match the consensus of the aligned sequences are boxed. The sequence of HXB2 was included as a reference.
(0.61 MB PDF)

Figure S2 Phylogenetic analysis of gp120. Phylogenetic relationships between the 10 subtype C gp120 genes selected for expression studies and 12 clade C genes from standard reference panels. Ten clade C genes were obtained from the UNAIDS and NIAID Networks for HIV Isolation and characterization. These included: ZA97002.7-7, ZA9710, ZA9712, CN97001, CN98005, IN98025, IN98026, TZ97005, TZ97008, ZM651. These were compared to 12 envelope genes from the subtype C reference panel of Li et al. [12] and 3 clade C reference sequences from the Los Alamos sequence database www.hiv.lanl.gov/.webloc. The tree was constructed in EMBOSS http://www.ebi.ac.uk using the neighbor joining method [29] by percentage identity, on sequences pre-aligned using the MAFFT algorithm [30]. Horizontal lengths are proportional to distance, vertical distances are for clarity only.
(0.04 MB PDF)

Figure S3 V3 PhylogeneticTree. Phylogenetic relationships between the the V3 domains of 10 subtype C gp120 genes selected for expression studies and the V3 domains of 12 clade C genes from standard reference panels. Ten clade C genes were obtained from the UNAIDS and NIAID Networks for HIV Isolation and characterization. These included: ZA97002.7-7, ZA9710, ZA9712, CN97001, CN98005, IN98025, IN98026, TZ97005, TZ97008, ZM651. These were compared to 12 envelope genes from the subtype C reference panel of Li et al. [12] and 3 clade C reference sequences from the Los Alamos sequence database www.hiv.lanl.gov/.webloc. The tree was constructed in EMBOSS http://www.ebi.ac.uk using the neighbor joining method [29] by percentage identity, on sequences pre-aligned using the MAFFT algorithm [30]. Horizontal lengths are proportional to distance, vertical distances are for clarity only.
(0.05 MB PDF)

Figure S4 Binding of antisera against subtype C gp120s to envelope proteins from clade C, B, E, and D viruses. ELISA plates were coated with the gp120 from subtype C (ZM651), B (MN), E (A244) or D (Z6). Pooled antisera raised against subtype C gp120s indicated were incubated at a single dilution (1∶5000), allowed to bind, then detected using an HRP-labeled anti-guinea pig antibody. OD490 for a single dilution is shown. The data bars are color coded and correspond to the following strains: ZM1, ZM651; IN1, IN98025; IN2, IN98026; TZ2, TZ97008; ZA1, ZA97002; TZ1, TZ97005; CN2, CN98005; ZA2, ZA97010; CN1, CN97001; ZA3, ZA97012.
(0.11 MB PDF)

Table S1 Sequence of V3 peptides used in ELISA. The sequence of V3 peptides from the MN (clade B) and A244 (clade E) strains of HIV are indicated, along with the sequences of peptides synthesized based on consensus sequences from clade A, C and D envelope proteins. The clade C consensus sequence matched that of the ZM651, CN97001, CN97005, and IN98026 strains of HIV. The IN1, TZ1, ZA1/ZA2, and ZA3 peptides matched the sequences of the IN98025, TZ97005, ZA97002, and ZA97012 envelopes, respectively. The consensus clade A V3 peptide matched that found in the TZ97008 isolate.
(0.08 MB PDF)
