# Supplementary material for: Comparative Immunogenicity of HIV-1 Clade C Envelope Proteins for Prime/Boost Studies
Source: PLoS One. 2010 Aug 11;5(8):e12076. doi: 10.1371/journal.pone.0012076 (PMC2920315; doi:10.1371/journal.pone.0012076)
Supplement: Figure S1 — Alignment of subtype C gp120 amino acid sequences. Amino acid sequences were deduced from the DNA sequences of the 10 subtype C gp120 genes. The sequences were aligned using the MacVector Sequence Analysis package (Accelrys Inc. San Diego, CA). The alignment was then edited manually to align significant features, e.g. a pair of conserved N-linked glycosylation sites in the V1 hypervariable region. Bases that match the consensus of the aligned sequences are boxed. The sequence of HXB2 was included as a reference. (0.61 MB PDF) [file pone.0012076.s001.pdf]

Supplemental Figure S1. Alignment of subtype C gp120 amino acid sequences.

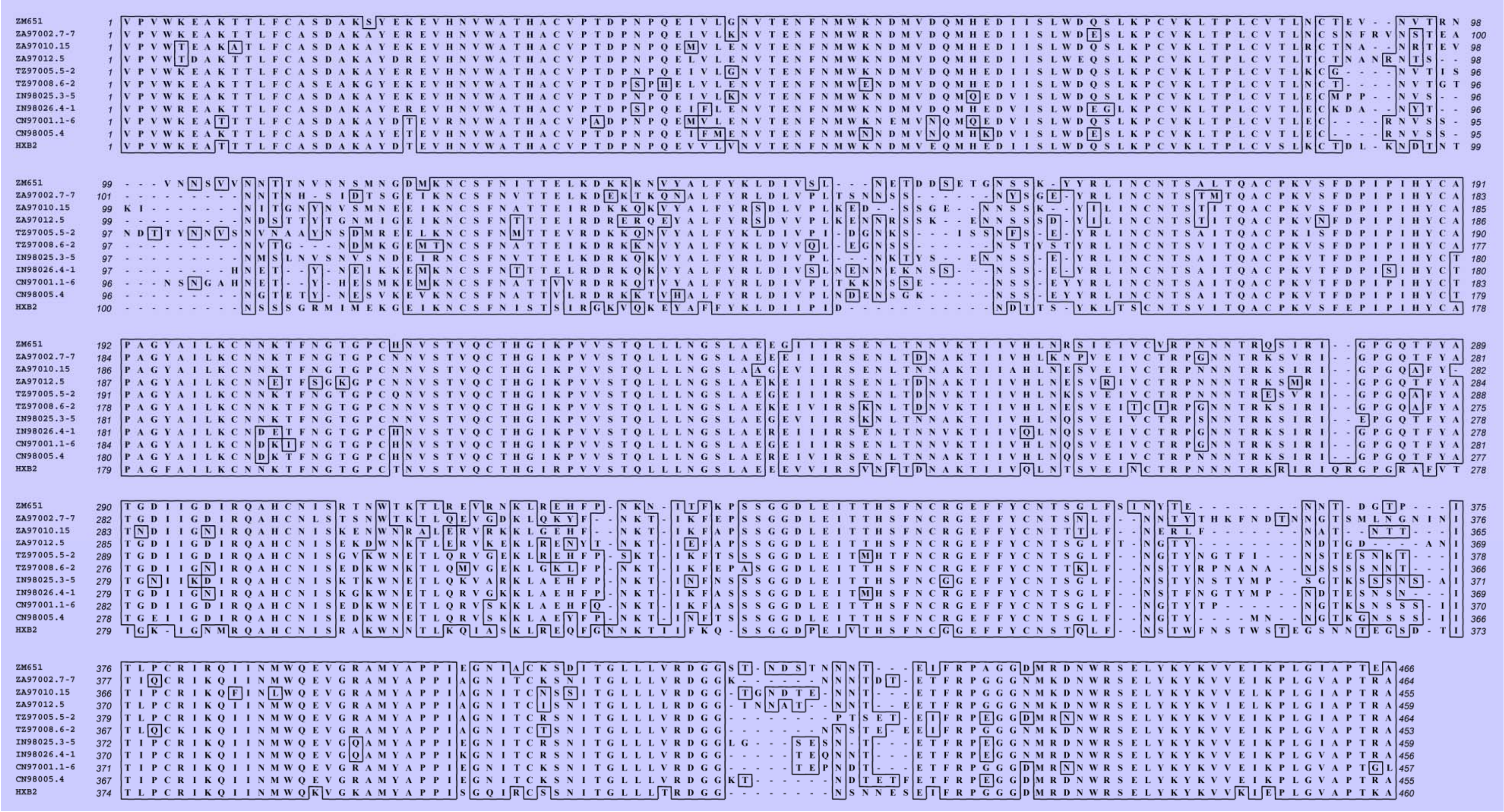

Amino acid sequences were deduced from the DNA sequences of the 10 subtype C gp120 genes. The sequences were aligned using the MacVector Sequence Analysis package (Accelrys Inc. San Diego, CA). The alignment was then edited manually to align significant features, e.g. a pair of conserved N-linked glycosylation sites in the V1 hypervariable region. Bases that match the consensus of the aligned sequences are boxed. The sequence of HXB2 was included as a reference.
