# Supplementary material for: Comparative Immunogenicity of HIV-1 Clade C Envelope Proteins for Prime/Boost Studies
Source: PLoS One. 2010 Aug 11;5(8):e12076. doi: 10.1371/journal.pone.0012076 (PMC2920315; doi:10.1371/journal.pone.0012076)
Supplement: Figure S2 — Phylogenetic analysis of gp120. Phylogenetic relationships between the 10 subtype C gp120 genes selected for expression studies and 12 clade C genes from standard reference panels. Ten clade C genes were obtained from the UNAIDS and NIAID Networks for HIV Isolation and characterization. These included: ZA97002.7-7, ZA9710, ZA9712, CN97001, CN98005, IN98025, IN98026, TZ97005, TZ97008, ZM651. These were compared to 12 envelope genes from the subtype C reference panel of Li et al. [12] and 3 clade C reference sequences from the Los Alamos sequence database www.hiv.lanl.gov/.webloc. The tree was constructed in EMBOSS http://www.ebi.ac.uk using the neighbor joining method [29] by percentage identity, on sequences pre-aligned using the MAFFT algorithm [30]. Horizontal lengths are proportional to distance, vertical distances are for clarity only. (0.04 MB PDF) [file pone.0012076.s002.pdf]

Supplemental Figure S2. Phylogenetic Analysis of gp120

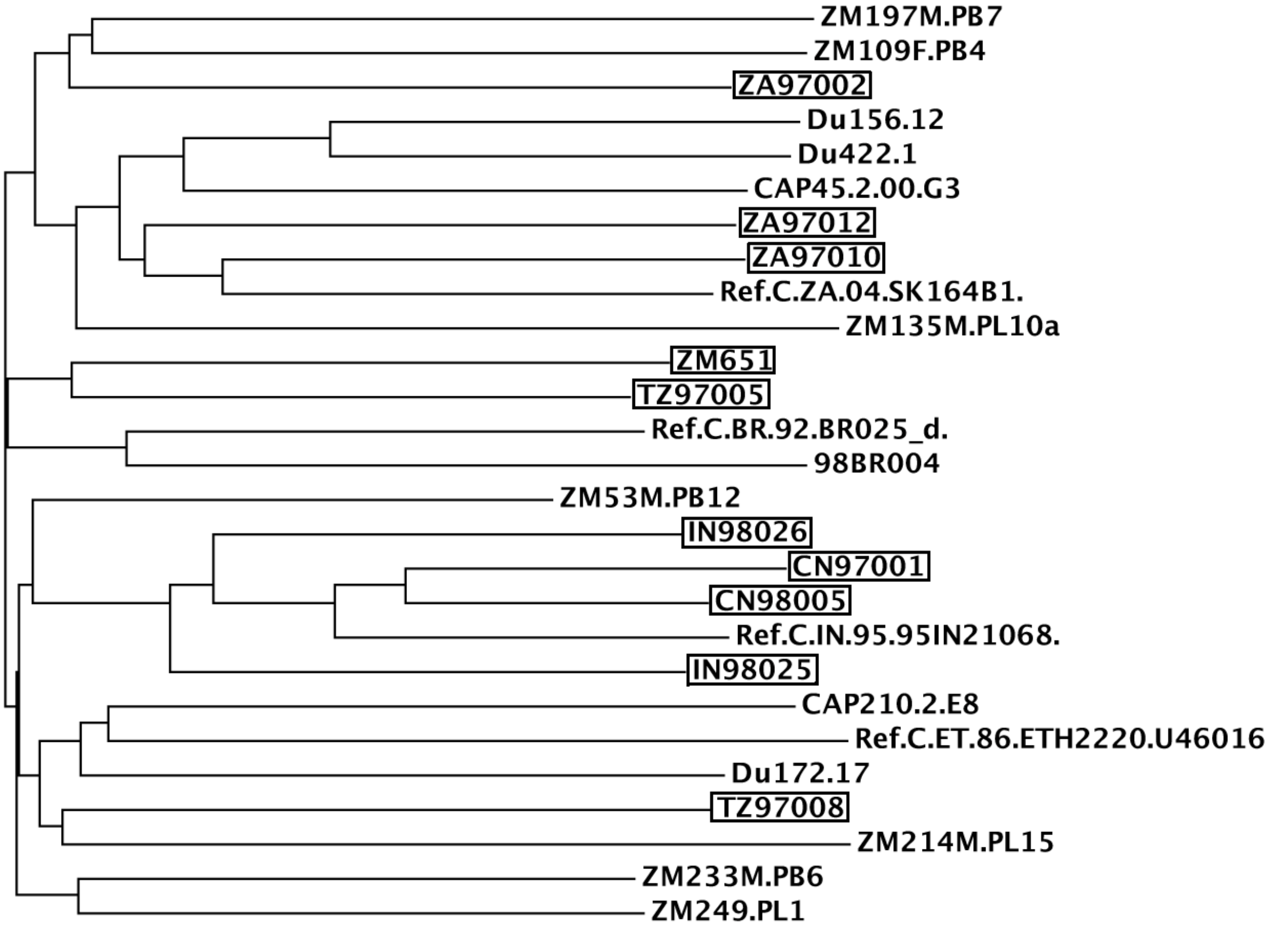

Phylogenetic relationships between the 10 subtype C gp120 genes selected for expression studies and 12 clade C genes from standard reference panels. Ten clade C genes were obtained from the UNAIDS and NIAID Networks for HIV Isolation and characterization. These included: ZA97002.7-7, ZA9710, ZA9712, CN97001, CN98005, IN98025, IN98026, TZ97005, TZ97008, ZM651. These were compared to 12 envelope genes from the subtype C reference panel of Li et al. [12] and 3 clade C reference sequences from the Los Alamos sequence database www.hiv.lanl.gov/.webloc. The tree was constructed in EMBOSS http://www.ebi.ac.uk using the neighbor joining method [29] by percentage identity, on sequences pre-aligned using the MAFFT algorithm [30]. Horizontal lengths are proportional to distance, vertical distances are for clarity only.
