# Supplementary material for: Comparative Immunogenicity of HIV-1 Clade C Envelope Proteins for Prime/Boost Studies
Source: PLoS One. 2010 Aug 11;5(8):e12076. doi: 10.1371/journal.pone.0012076 (PMC2920315; doi:10.1371/journal.pone.0012076)
Supplement: Figure S4 — Binding of antisera against subtype C gp120s to envelope proteins from clade C, B, E, and D viruses. ELISA plates were coated with the gp120 from subtype C (ZM651), B (MN), E (A244) or D (Z6). Pooled antisera raised against subtype C gp120s indicated were incubated at a single dilution (1∶5000), allowed to bind, then detected using an HRP-labeled anti-guinea pig antibody. OD490 for a single dilution is shown. The data bars are color coded and correspond to the following strains: ZM1, ZM651; IN1, IN98025; IN2, IN98026; TZ2, TZ97008; ZA1, ZA97002; TZ1, TZ97005; CN2, CN98005; ZA2, ZA97010; CN1, CN97001; ZA3, ZA97012. (0.11 MB PDF) [file pone.0012076.s004.pdf]

Supplemental Figure S4. Binding of antisera against subtype C gp120s to envelope proteins from clade C, B, E, and D viruses.

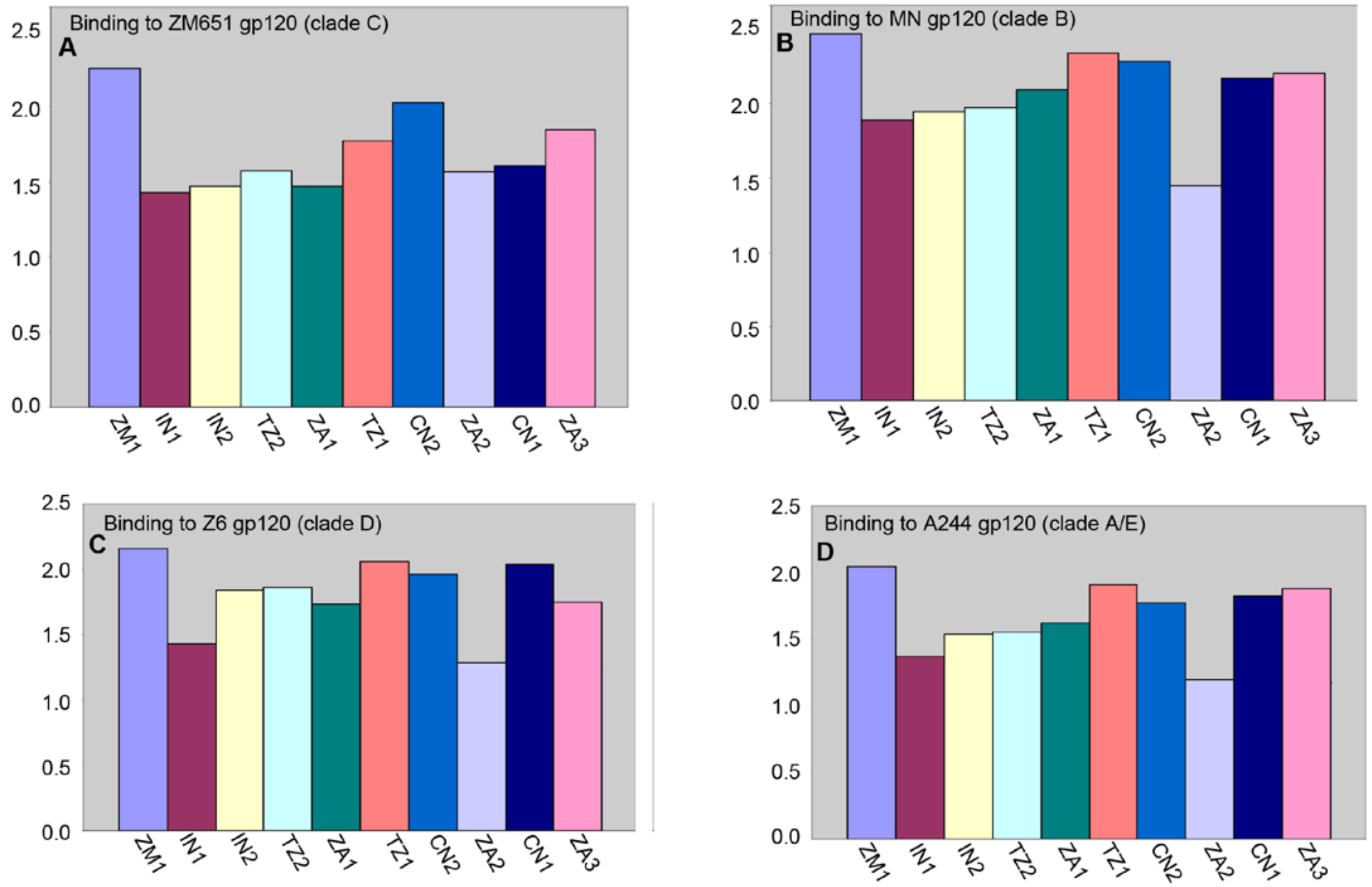

ELISA plates were coated with the gp120 from subtype C (ZM651), B (MN), E (A244) or D (Z6). Pooled antisera raised against subtype C gp120s indicated were incubated at a single dilution (1:5000), allowed to bind, then detected using an HRP-labeled anti-guinea pig antibody. OD490 for a single dilution is shown. The data bars are color coded and correspond to the following strains: ZM1, ZM651; IN1, IN98025; IN2, IN98026; TZ2, TZ97008; ZA1, ZA97002; TZ1, TZ97005; CN2, CN98005; ZA2, ZA97010; CN1, CN97001; ZA3, ZA97012.
