# Supplementary material for: Comparative Immunogenicity of HIV-1 Clade C Envelope Proteins for Prime/Boost Studies
Source: PLoS One. 2010 Aug 11;5(8):e12076. doi: 10.1371/journal.pone.0012076 (PMC2920315; doi:10.1371/journal.pone.0012076)
Supplement: Table S1 — Sequence of V3 peptides used in ELISA. The sequence of V3 peptides from the MN (clade B) and A244 (clade E) strains of HIV are indicated, along with the sequences of peptides synthesized based on consensus sequences from clade A, C and D envelope proteins. The clade C consensus sequence matched that of the ZM651, CN97001, CN97005, and IN98026 strains of HIV. The IN1, TZ1, ZA1/ZA2, and ZA3 peptides matched the sequences of the IN98025, TZ97005, ZA97002, and ZA97012 envelopes, respectively. The consensus clade A V3 peptide matched that found in the TZ97008 isolate. (0.08 MB PDF) [file pone.0012076.s005.pdf]

Supplemental Table S1. Sequence of V3 peptides used in ELISA

MN	N K R K R I H I G P G R A F Y T T K N I I G T I
Consensus D	N T R Q R T P I G L G Q A L Y T T R G I I G D I
A244	N T R T S I T I G P G Q V F Y R T G D I I G D I
Consensus C/ZM1/CN1/CN2/I	N T R Q S I R I G P G Q T F Y A T G D I I G D I
Consensus A/TZ2	N T R K S I R I G P G Q A F Y A T G D I I G D I
IN1	N T R K S I R I E P G Q T F Y A T G N I I K D I
TZ1	N T R E S V R I G P G Q A F Y A T G D I I G D I
ZA1/ZA2	N T R K S V R I G P G Q T F Y A T G D I I G D I
ZA3	N T R K S M R I G P G Q T F Y A T G D I I G D I

The sequence of V3 peptides from the MN (clade B) and A244 (clade E) strains of HIV are indicated, along with the sequences of peptides synthesized based on consensus sequences from clade A, C and D envelope proteins. The clade C consensus sequence matched that of the ZM651, CN97001, CN97005, and IN98026 strains of HIV. The IN1, TZ1, ZA1/ZA2, and ZA3 peptides matched the sequences of the IN98025, TZ97005, ZA97002, and ZA97012 envelopes, respectively. The consensus clade A V3 peptide matched that found in the TZ97008 isolate.
